# Secondary Immune Thrombocytopenia (ITP) Associated with ChAdOx1 Covid-19 Vaccination – A Case Report

**DOI:** 10.1055/s-0041-1731774

**Published:** 2021-07-30

**Authors:** Martin Koch, Sybille Fuld, Jan M. Middeke, Julia Fantana, Simone von Bonin, Jan Beyer-Westendorf

**Affiliations:** 1 Department of Medicine, Hematology Division, University Hospital Carl Gustav Carus, Dresden, Germany; 2 Department of Medicine, Nephrology Division, University Hospital Carl Gustav Carus, Dresden, Germany; 3 Department of Medicine, Emergency and Intensive Medicine Division, University Hospital Carl Gustav Carus, Dresden, Germany

**Keywords:** thrombocytopenia, platelet pathology / inherited, aquired, infectious diseases, Covid Vaccination, ITP, ITP associated with Covid vaccination, ITP associated with AstraZeneca Covid vaccination, secondary ITP

## Abstract

Novel mRNA and vector-based covid-19 vaccinations have shown high efficacy in preventing symptomatic COVID-19 infections. Compared with the number of performed vaccinations, rates of severe side effects seem low. Rare prothrombotic coagulation disorders with suspected association to ChAdOx1 nCoV-19 (AstraZeneca) have been reported. These cases have gathered considerable media attention and caused a temporary pause of usage of the AstraZeneca vaccine in Europe and several other countries and are currently discussed as vaccine-induced immune thrombotic thrombocytopenia (VITT). However, hemorrhagic complications from ChAdOx1 nCoV-19 vaccination have also been reported but, so far, received less public attention despite considerable potential for life-threatening complications. Here we present a case of severe immune thrombocytopenia after ChAdOx1 covid-19 vaccination and its successful primary management.

## Case Report

A 41-year-old caucasian male presented to the emergency department in March 2021 with petechial and mucosal bleeding. Blood tests revealed a severe thrombocytopenia with platelet count of <1GPt/L and normal hemoglobin and leucocyte levels with no further laboratory abnormalities in routine tests. Screening for SARS-CoV2-infection was negative.

Closer review of the patient's medical history revealed that he had received the first dose of ChAdOx1 Covid-19 vaccination (AstraZeneca) 14 days prior to hospital admission. Apart from a severe pneumonia as a child and knee joint surgery several years ago, there were no prior medical conditions, as well as no long-term medication. The patient did not have a suggestive bleeding history and never experienced any petechiae. The last blood sample was taken in January of 2013 revealing a platelet count of 189 Gpt/L. Immediately after application of the vaccine, the initial reaction was mild and limited to swelling and pain at the injection site. The patient reported an episode of severe headache combined with blurry vision ∼8 days post vaccination, leading to a head MRI showing no pathologic findings. First petechial bleedings occurred 10 days, mucosal bleeding 11 days after vaccination.

On the evening of admission, thrombotic thrombocytopenia purpura (TTP) was ruled out by normal neurological and kidney function, absence of fragmentocytes, and regular ADAMTS13 activity. 1 Plasma d-dimer levels (<270 ng/ml FEU) and plasma fibrinogen levels (2,66 g/L) were in the range of normal.

Additional ELISA (enzyme linked immunosorbent assay) testing for platelet factor 4 antibodies (AcuStar; IL Werfen®) and functional heparin-induced platelet activation test (HIPA; APACT 4s PLUS; BioChemica®) excluded heparin-induced thrombocytopenia (HIT). Based on an established collaboration we were able to immediately obtain additional analyses from the reference laboratory in Greifswald, Germany, ruling out vaccine-induced immune thrombotic thrombocytopenia (VITT) using a modified HIPA assay. 2 Our diagnostic approach therefore was in line with the later published guidance from the German scientific society of thrombosis- and hemostasis (GTH) on vaccination with the AstraZeneca Covid 19 vaccine 3 and the recommendations for the clinical and laboratory diagnosis of VITT by the ISTH SSC Subcommittee on Platelet Immunology. 4

We tested for several other conditions that are known to cause secondary ITP. Antiphospholipid syndrome was ruled out by normal test results for lupus-anticoagulant and IgG/IgM antibodies to cardiolipin and β2-glycoprotein-I. Testing for hepatitis B and C, HIV, and H. pylori was also negative.

After exclusion of these causes for isolated severe thrombocytopenia, clinical manifestation (acute onset of severe thrombocytopenia, typical bleeding pattern) was suggestive for immune thrombocytopenia (ITP). Due to the suggestive temporal connection, we concluded that ChAdOx1 Covid-19 vaccination was the most likely trigger for this case of secondary ITP. Treatment with glucocorticoids (prednisone 2 mg/kg bodyweight once daily) and a single dose of intravenous immunoglobulin (IVIG) (1 g/kg body weight) was initiated within 2 hours after admission. Platelet levels gradually increased to 36 Gpt/L within 60 hours with concomitant improvement of bleeding symptoms. The patient was discharged on day 4. Further treatment was conducted by an outpatient hematologist. A pause in corticoid therapy right after discharge caused a drop in platelets (to 14 Gpt/L on day 3 after discharge) – oral prednisolone therapy was restarted and, due to an unsatisfactory rise in platelets, the patient received two further doses of IVIG (0,4 g/kg body weight) from his outpatient hematologist on days eight and ten after discharge. Upon last contact (25 days after discharge – 29 days after first treatment) platelet levels were 80 Gpt/l with a daily dexamethasone doses of 24 mg and a plan for tapering.

ITP is a rare autoimmune disorder with reduced numbers of circulating platelets and occasionally impaired megakaryopoiesis. In some cases (auto-) antibodies against platelet antigens can be detected. Etiology of ITP can be primary - without any obvious cause (formerly termed idiopathic thrombocytopenic purpura) - or secondary, meaning ITP in association with malignant, infectious or autoimmune diseases, or drugs. The overall incidence of ITP varies from 2,9 to 3,3/100.000 person-years with peaks among children and in people >60 years of age with a slightly higher rate in females than in males. 5 6 Vaccine-associated ITPs contribute to the overall incidence with ∼1% of all ITP cases. 7

ITP is a known side effect especially of vaccination against influenza, 8 but also measles-mumps-rubella (MMR), haemophilus influenza and hepatitis B virus (HBV). 9

ChAdOx1 nCoV-19 is a replication-defective chimpanzee adenovirus-vectored vaccine. It is genetically modified to express the full-length COVID-19 spike glycoprotein gene. *ChAdOx1 nCoV-1* 9 is given as two intramuscular injections 4 to 12 weeks apart. It received conditional marketing authorization in the European Union on 29th of January 2021.

A recent interim analysis of the Oxford COVID Vaccine Trial Group reported an incidence of serious adverse events (SAE) of 108 of 12282 participants (0.9%) in the ChAdOx1 nCoV-19 vaccination group. 10 No serious cases of blood or lymphatic system disorders were described. In the prescribing information, lymphadenopathy is listed as the only hematological side effect. In a press release on 14 ^th^ of March 2021, the manufacturer declared a lack of evidence for an increase of major bleeding events in over 60.000 participants enrolled in clinical trials. 11

By 18 ^th^ of March 2021, ∼20 million individuals in the UK and EEA have been vaccinated. By 22 ^nd^ of March 2021, the *European database of suspected adverse drug reaction reports* listed 1867 cases of blood and lymphatic system disorders with a temporal association to *ChAdOx1* vaccination, including 128 cases with thrombocytopenia or bleeding tendency. 12 In addition 7 cases of disseminated intravascular coagulation (DIC), 18 cases of cerebral venous sinus thrombosis (CVST; 9 cases resulting in death) and an unspecified number of major bleeding complications associated with thrombocytopenia were reported. 13 Several case series involving patients with acute thrombocytopenia and thrombosis in unusual localizations (e.g., CVST, internal jugular vein thrombosis, portal vein thrombosis, etc.) were published, 2 14 15 in most of the further investigated cases, patient serum showed strong reactivity (platelet activation) on PF4 ELISA, confirmed with a modified heparin-induced platelet aggregation assay (HIPAA) despite a lack of heparin exposure, supporting an autoimmunological cause for the thrombotic vaccination complications (HIT mimicry). 2 The authors suggest naming this novel entity vaccine-induced immune thrombotic thrombocytopenia (VITT). However, on 31 ^th^ of March 2021, the European Medicines Agency (EMA) publicly announced that analysis of the rare SAE such as CSVT, DIC and thrombotic events showed insufficient evidence to support an increased risk associated with ChAdOx1 nCoV-19 vaccination. 16

Contrary to the well documented cases of VITT, case reports on ITP associated with ChAdOx1 vaccination have not yet been published in scientific journals. The two entities are clinically distinguishable by the lack of major thrombotic events in unusual localizations in ITP patients.

However, cases of ITP associated with Covid-19 mRNA vaccine (manufactured by Pfizer/BioNTech and Moderna) have gained public awareness recently. The case of a Florida doctor dying from ITP has received intensive media coverage 17 two case reports have been reported in scientific journals. 18 19 In their commentary in the AJH Lee et al. list 17 cases if ITP of following mRNA vaccination that have been reported in the US Vaccine Adverse Events Reporting System (VAERS). The authors suggest, that mRNA vaccines – especially the outer lipid layer of nanoparticles – cannot be ruled out as a trigger for de novo ITP. 20

Our case, in context with the above mentioned (and daily changing) pharmacovigilance data illustrates, at least four important clinical management aspects:

1.) Adverse autoimmunologic reactions are rare, but potentially life-threatening clinical manifestations, manifesting as early as within two first weeks after COVID-19 vaccination. This observation warrants inclusion in patient information material, including guidance to raise awareness for neurologic and hemorrhagic symptoms.
2.) Although first reports were related to thrombotic manifestations after ChAdOx1 nCoV-19 vaccine, it can be expected that such autoimmunologic reactions can also occur with other COVID-19 vaccines especially those using viral vectors. A careful surveillance and transparent reporting of such events is essential to update-risk-benefit assessments by health authorities and health care providers.
3.) Reported adverse autoimmunological reactions to COVID-19 vaccines include thromboembolic and hemorrhagic conditions. This distinctive difference is important to report to the medical community in detail to prevent overuse of anticoagulant drugs under the misconception that vaccines generally increase the risk for thromboembolism. Cases of ITP or TTP might be rare but it can be expected that unwarranted thromboprophylaxis would contribute to life-threatening bleeding. Furthermore, occult patient-related bleeding disorders may exacerbate from unwarranted anticoagulant treatments.
4.) So far, no predisposing factors for adverse autoimmunologic reactions to COVID-19 vaccines have been identified. Given the global scale of COVID-19 and the need to provide vaccines to billions of patients, intensified efforts are needed to establish risk prediction models and early detection measures to reduce incidence and mortality of these drug-associated major complications.

Although the present case of ITP was severe and a causal association to the vaccine is plausible, vaccination against the novel corona virus is highly recommended. SARS-CoV2-infections itself are known to cause ITP 21 with a relatively high rate of bleeding events. 22 Established therapies (corticosteroids and IVIG) have proven to be effective in treating ITP irrelevant to its cause. Treatment with Rituximab should be carefully evaluated due to possible slow reaction and impairment of the response to the vaccination. 20

With established risk prediction models, derived preventive measures and early and effective treatment of adverse autoimmunologic reactions the public support for a broad vaccination campaign could be restored, replacing the current situation of mistrust and misinformation.

## Main Concepts and Learning Points

– Despite public focus on thromboembolic complications from COVID vaccines other autoimmune complications such as autoimmune thrombocytopenia or thrombotic microangiopathy are not uncommon
– We present a case with thrombocytopenia after vaccination with ChAdOx1 covid-19 vaccination (AstraZeneca), clinically manifesting with mucosal and petechial skin bleeding and severe headache
– Standard therapy with corticosteroids and IVIG proved to be an effective and save treatment
– a consequent diagnostic workup of manifest thrombocytopenia following COVID-19 vaccination is important to differentiate prothrombotic states (such as heparin-induced thrombocytopenia or disseminated intravascular coagulation) from autoimmune thrombocytopenia
